# Rapid and visual detection of *Lawsonia intracellularis* with an improved recombinase polymerase amplification assay combined with a lateral flow dipstick

**DOI:** 10.1186/s12917-019-1841-9

**Published:** 2019-03-21

**Authors:** Yanyang Wu, Kaiyue Tian, Yuhan Zhang, Huifang Guo, Ning Li, Zeng Wang, Jun Zhao

**Affiliations:** College of Animal Science and Veterinary Medicine, Henan Agricultural University, Zhengzhou, Henan China

**Keywords:** *Lawsonia intracellularis*, Recombinase polymerase amplification, Lateral flow dipstick, On-site detection

## Abstract

**Background:**

*Lawsonia intracellularis (L. intracellularis)* is the etiologic agent of porcine proliferative enteropathy (PPE), which is reported in many swine breeding countries all over the world, and has caused enormous economic losses in intensive pig production systems. Therefore, the aim of this study was to develop a simple and rapid method for on-site detection of *Lawsonia intracellularis* (*L. intracellularis*). As the isothermal recombinase polymerase amplification (RPA) can be performed at a constant temperature and its product is directly observed on a lateral-flow dipstick (LFD) with naked eyes without electrophoresis, the RPA-LFD assay should be useful for field diagnosis of *L. intracellularis* as well as its detection from clinical samples.

**Results:**

The established RPA-LFD assay could be finished in 30 min at a wide temperature range of 25 to 40 °C, and the amplicons could be visualized by naked eyes. The developed RPA-LFD assay was specific to dnaA gene of *L. intracellularis*, and did not detect nucleic acids extracted from other common gastrointestinal pathogens. The minimum detection of this RPA-LFD method was 400 *L. intracellularis* per reaction, which was as sensitive as conventional PCR. Further, the RPA-LFD assay was performed with 150 clinical fecal samples and the detection results were compared with conventional PCR. Results showed that the coincidence rate of RPA-LFD and conventional PCR was 100%.

**Conclusions:**

The combined RPA with LFD assay provides a simple, rapid, specific and sensitive alternative for field diagnosis of *L. intracellularis* infection.

## Background

*Lawsonia intracellularis*, a fastidious and obligate intracellular bacterium, belongs to the bacterial genus *Lawsonia* and is the etiologic agent of porcine proliferative enteropathy (PPE), which is characterized clinically by mild to severe diarrhea, retarded growth and/or sudden death in fattening pigs and macroscopically by thickening of the intestinal mucosa due to enterocyte proliferation [1, 2]. Since the first case of *L. intracellularis* infection was reported in 1931 [3], the prevalence of PPE has been reported in many swine breeding countries all over the world, and has caused enormous economic losses in intensive pig production systems [4–9]. In China, *L. intracellularis* was first isolated from the intestinal mucosa of infected pigs in Southern China in 2008 [10]. The overall seroprevalence of *L. intracellularis* in Chinese pig herds was 77%, evaluated by a blocking ELISA in 2014. A higher seroprevalence was found in fattening pigs, sows and boars compared with pre-weaning piglets and weaners [11]. To control the infection and spread of the *L. intracellularis* effectively, simple, rapid, sensitive and accurate methods suitable for field detection of *L. intracellularis* are critical.

The diagnosis of *L. intracellularis* is based on the demonstration of the microbe or microbial DNA in tissue specimens or fecal samples, or the demonstration of *L. intracellularis*-specific antibodies in sera. Current available diagnostic methods for *L. intracellularis* infection in live animals include serological tests for detecting antibody against *L. intracellularis*, such as enzyme-linked immunosorbent assay (ELISA) [12–14], immunoperoxidase monolayer assay (IPMA) [15], indirect immunofluorescence assay (IFA) [16], and methods for detecting *L. intracellularis* in fecal samples, such as polymerase chain reaction (PCR)-based tests [17–24] and IFA [25]. Among these methods, bacteria maintenance in vitro has limited the use of serological tests, since the cultivation of the obligate intracellular *L. intracellularis* requires establishment of suitable cell lines and *L. intracellularis* cannot be cultured in conventional cell-free medium. Although PCR-based molecular diagnostic tools are sensitive and capable of detecting *L. intracellularis* from various clinical samples, these techniques are restricted to the laboratory due to the requirements for sophisticated equipment, complex experimental procedures, skilled personnel and subsequent analysis. PPE may also be diagnosed postmortem from the typical macroscopic lesions, but histological confirmation by immunohistochemistry (IHC) test is needed [26–28]. Since *L. intracellularis* infection does not show specific clinical signs of illness at the earlier stage of infection, which often leads to the missing diagnosis. Hence there is an urgent need for a rapid and reliable pen side diagnostic assay for a better detection and control of this economically important disease of swine.

Recombinase polymerase amplification (RPA) is a novel isothermal amplification technology which couples isothermal recombinase-driven primer targeting of template material with strand-displacement DNA synthesis [29]. It overcomes the technical difficulties posed by current DNA amplification methods. RPA does not require a stringent incubation temperature for optimal performance and can achieve exponential amplification without pretreatment of sample DNA. The RPA reactions are sensitive, specific, rapid and applicable at constant low temperature ranging from 25 to 43 °C [30]. Lateral flow dipstick (LFD) is a portable diagnostic tool which can be used for detecting various kinds of substances in non-laboratory situations. The workflow does not require highly qualified personnel and the results can be inspected by the naked eyes. LFD has been successfully incorporated into various nucleic acids based methods [31–35]. This unique combination of properties is a significant advance in the development of portable and widely accessible nucleic acid-based tests.

In this study, RPA coupled with LFD system (RPA-LFD) was developed to detect *L. intracellularis* DNA in fecal samples for the first time. The specificity and sensitivity of the RPA-LFD were evaluated. And the effectiveness of the RPA-LFD was determined by detecting clinical fecal samples.

## Results

### Usability of the primers

**Fig. 1:**
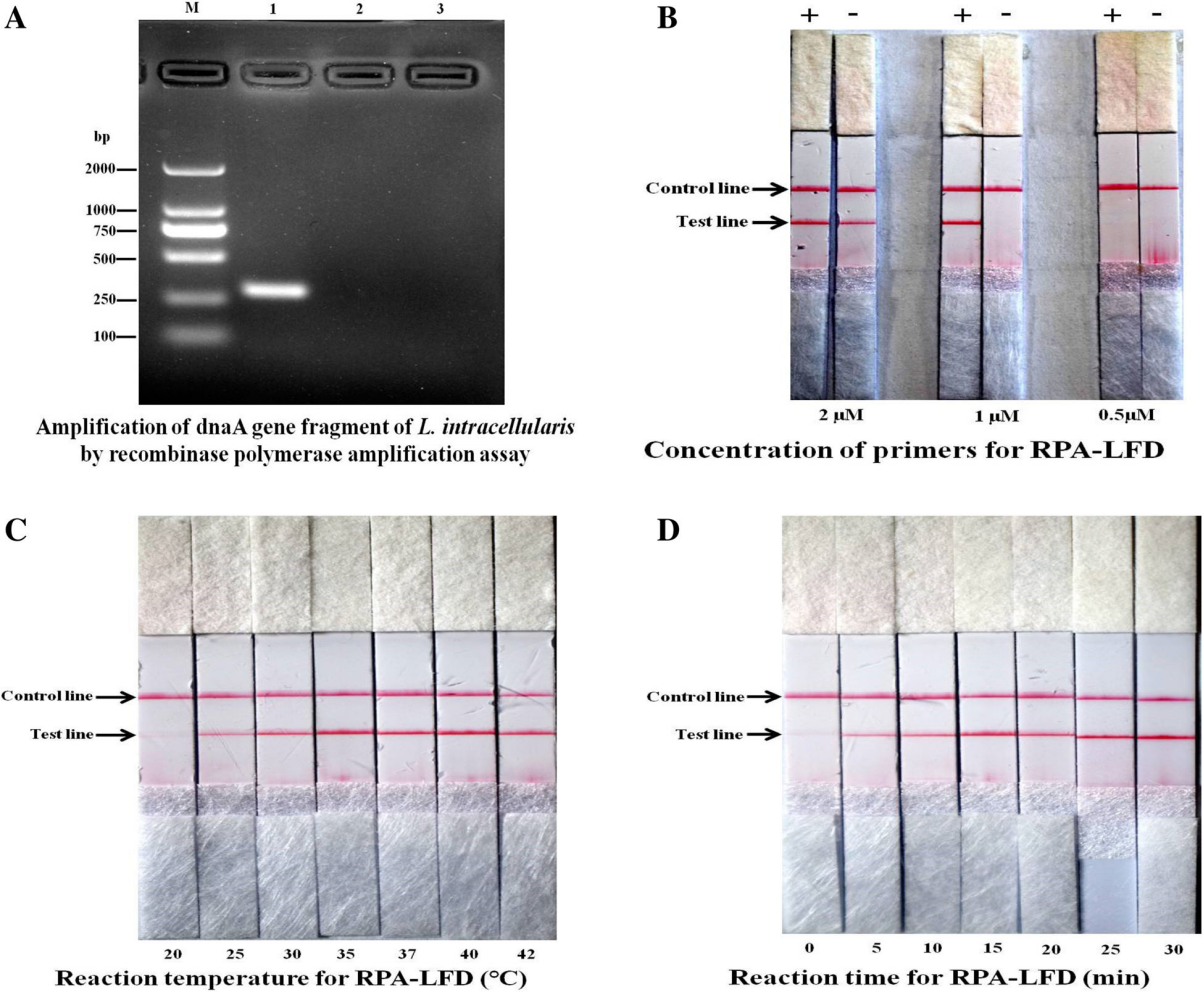
Specific amplification of dnaA gene and optimization of condition for *L. intracellularis* RPA-LFD. The dnaA gene fragment of *L. intracellularis* was specifically amplified by recombinase polymerase amplification (RPA) using designed primers (**a**); Concentration of primers (**b**), reaction temperature (**c**), and reaction time (**d**) were optimized for RPA, and the amplicons were detected using lateral flow dipstick (LFD). M: DL2000 DNA Marker; 1: *L. intracellularis* genomic DNA; 2: *E. coli* genomic DNA; 3: Blank control. *L. intracellularis* DNA positive samples (+) and negative samples (−) were used as templates in RPA, respectively

The specificity of the RPA primers was determined by BLAST search (http://www.ncbi.nlm.nih.gov/blast/Blast.cgi). As expected, the primer combination was 100% identical to sequences of dnaA gene of *L. intracellularis* from pigs (GenBank accession nos. CP004029 and AM180252). The RPA basic reaction was performed by using unlabeled primers at 37 °C for 25 min to test the usability of the primers. The result of amplification showed that the 292-bp target gene could be successfully amplified. The sequencing results of RPA products were in good agreement with the dnaA gene of *L. intracellularis*. However, the target gene could not be amplified from DNA sample negative for *L. intracellular*is and a non-template control (Fig. 1A).

### Optimal reaction conditions for RPA-LFD assay

For the adaptation to the LFD detection system, a labeling RPA was carried out. Instead of labeling lateral flow probe, a pair of labelled forward and reverse primers was used. The sequences of labelled primers were identical to those of the RPA basic primers except for containing FITC and Biotin at the 5′ end of the forward and reverse primer, respectively.

Based on the RPA basic reaction results, the optimal conditions of combining the RPA with lateral flow detection were determined with a consideration on the Test line signal strength, sensitivity and no Test line for *L. intracellularis* negative sample. The optimal concentration of labelled primers was determined first. The results of LFD detection showed that amplification with 1.0 μM of each labelled forward and reverse primer yielded the strongest Test line for *L. intracellularis* positive sample and no Test line for *L. intracellularis* negative sample (Fig. 1B).

By using the optimized primer concentration, the RPA temperature and reaction time were determined. Results of the amplification showed that, within the temperature range of 30–42 °C, the *L. intracellularis* DNA could be successfully amplified, and the optimal temperature range for the RPA was 37–42 °C (Fig. 1C). The routine temperature of 37 °C was chosen as the optimal RPA-LFD temperature.

Regarding the RPA reaction time, the results of LFD detection showed that the amplification signals appeared at 5 min after the start of the reaction. Amplifications for 5–30 min all yielded single and stable Test bands, and 15 min was chosen as the optimal RPA-LFD reaction time (Fig. 1D).

### Specificity of RPA-LFD assay

**Fig. 2:**
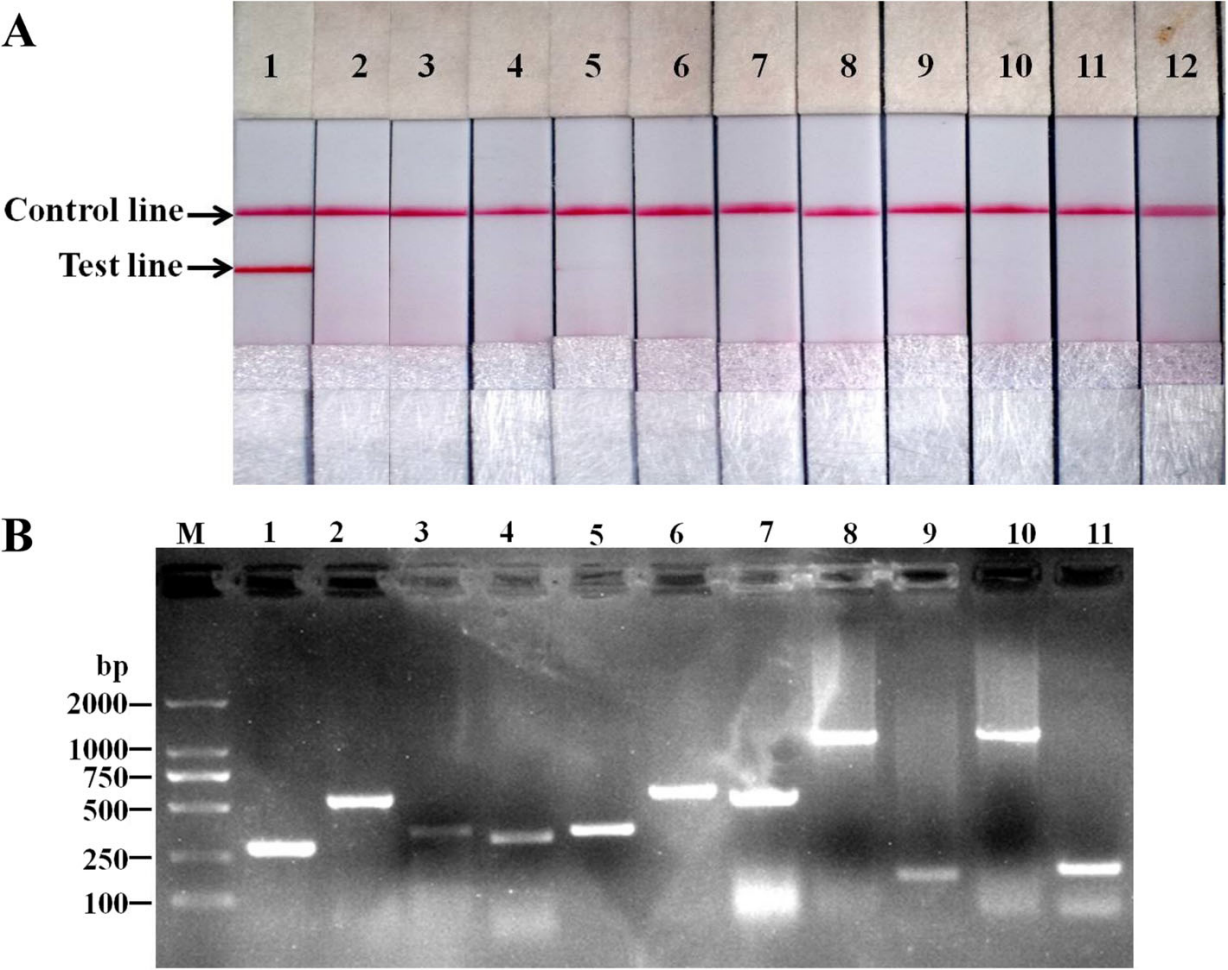
Specificity of the RPA-LFD assay. **a** The analytical specificity test demonstrated that DNAs from *E. coli*, *Salmonella* Cholerasuis (*S*. Cholerasuis), *Enterococcus faecalis* (*E. faecalis*), *Brachyspira hyodysenteriae* (*B*. *hyodysenteriae*), porcine circovirus serotype 2 (PCV2), pseudorabies virus (PRV) and porcine rotavirus (PoRV), and RNAs from porcine epidemic diarrhea virus (PEDV), porcine transmissible gastroenteritis virus (TGEV), classical swine fever virus (CSFV), and sample containing the above pathogens expect for *L. intracellularis* showed no cross-reactions in the developed *L. intracellularis* RPA-LFD (Lane 2–12), only sample containing both *L. intracellularis* and the above pathogens produced positive Test line (Lane 1); **b** Specific amplification *L. intracellularis*, *E. coli*, *S*. Cholerasuis, *E. faecalis*, *B. hyodysenteriae*, PCV2, PRV, PoRV, PEDV, TGEV, and CSFV by PCR using nucleic acids extracted from sample containing the above pathogens (Lane 1–12)

In order to evaluate the specificity of the *L. intracellularis*-specific RPA-LFD, the RPA was carried out by using 10 ng nucleic acids from each of the most common porcine gastrointestinal pathogens, including *E. coli*, *S.* Cholerasuis, *E. faecalis*, *B*. *hyodysenteriae*, PCV2, PRV, PoRV, PEDV, TGEV, CSFV, samples containing 10 ng nucleic acid of each above pathogens except for nucleic acid of *L. intracellularis* as well as a mixture of 10 ng nucleic acid of *L. intracellularis* and 10 ng nucleic acid of each above pathogens as templates, and the amplicons were detected by the LFD. As shown in Fig. 2A, except for DNA extracted from a mixture of *L. intracellularis* and the above pathogens, only red color Control line was observed on the LFD strips. The results showed that the RPA-LFD could discriminate *L.intracellularis* from other common porcine gastrointestinal pathogens. The existence of *L.intracellularis* and other common porcine gastrointestinal pathogens in the mixed sample was demonstrated by PCR using primer sets in Table 1 (Fig. 2B).

**Table 1:** Primer sequences for common porcine gastrointestinal pathogens

Pathogens	primers(5′-3′)	product length (base pair)	GenBank accession no.
*L. intracellularis*	F:AAATCCAAAAGTCGAGTATCTAACTGCGG	292	AM180252
	R:TAAAAACCCAGAGCAAAATCGTGATACCAGGCG		
*E. coli*	F:AACGGTTGCTCTTCATTTAG	678	CP034794
	R:GACCATCCAATAAGTGTG		
*S.* Cholerasuis	F:GCTCTTTCGTCTGGCATTA	351	CP034819
	R:AACTTCATCGCACCGTCA		
*E. faecalis*	F:AAAGTAGAATTAGATCCACAC	320	CP031027
	R:TCTATCACATTCGGTTGCG		
*B. hyodysenteriae*	F:ACTAAAGATCCTGATGTATTTG	352	CP019600
	R:CTAATAAACGTCTGCTGC		
PCV2	F:GGTGCCCGCTGCCACATCGAGAAAG	589	MH718995
	R:AGCTTCTACAGCTGGGACAGCAG		
PRV	F:GAGTACGTCACGGTCATCAAGGAG	553	NC006151
	R:CACTTCCGGTTTCTCCGGATC		
PoRV	F:GGCTTTAAAAGAGAGAATTTCCG	1062	JF835112
	R:GGTCACATCATACAATTCTAA		
PEDV	F:CATGGGCTAGCTTTCAGGTC	182	MK288006
	R:CGGCCCATCACAGAAGTAGT		
TGEV	F:TCTTTCTTCTACCCTATTATGACTG	1117	KT696544
	R:CTYTGGAGTATGGTGGTGTTC		
CSFV	F:GGTGGTCTAAGTCCTGAGTACAGG	177	MK121886
	R:GCCTCTGCAGCACCCTATCAGGTCG		

### Sensitivity of RPA-LFD assay

**Fig. 3:**
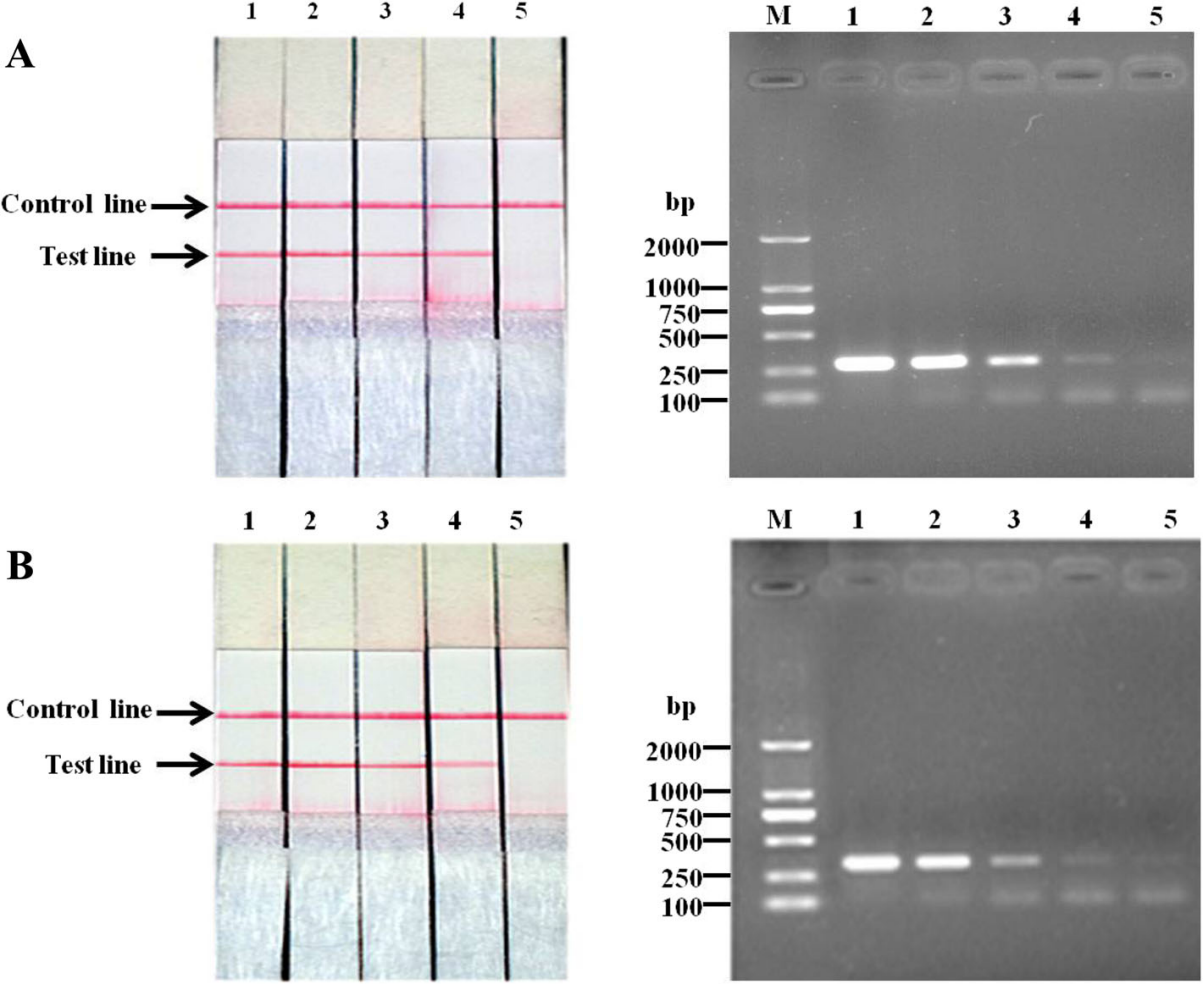
The analytical sensitivity of the RPA-LFD assay was evaluated and compared with conventional PCR by using genomic DNA extracted from pure-cultured and number-determined *L. intracellularis* (**a**) as well as feces sample spiked with pure-cultured and number-determined *L. intracellularis* (**b**) as templates. Lane 1–5: 4 × 10^5^–4 × 10^1^ bacteria per reaction. M: DL2000 DNA Marker

The analytical sensitivity of the RPA-LFD was determined by using genomic DNA extracted from pure-cultured and number-determined *L. intracellularis* and feces sample spiked with pure-cultured and number-determined *L. intracellularis*. As shown in Fig. 3, the established RPA-LFD had a same detection threshold of 400 *L. intracellularis* per reaction for both pure-cultured *L. intracellularis* and feces sample spiked with pure-cultured *L. intracellularis*. When the sensitivity of the RPA-LFD was compared with that of conventional PCR, the RPA-LFD was found as sensitive as the conventional PCR.

### Clinical performance of *L. intracellularis* RPA-LFD

**Fig. 4:**
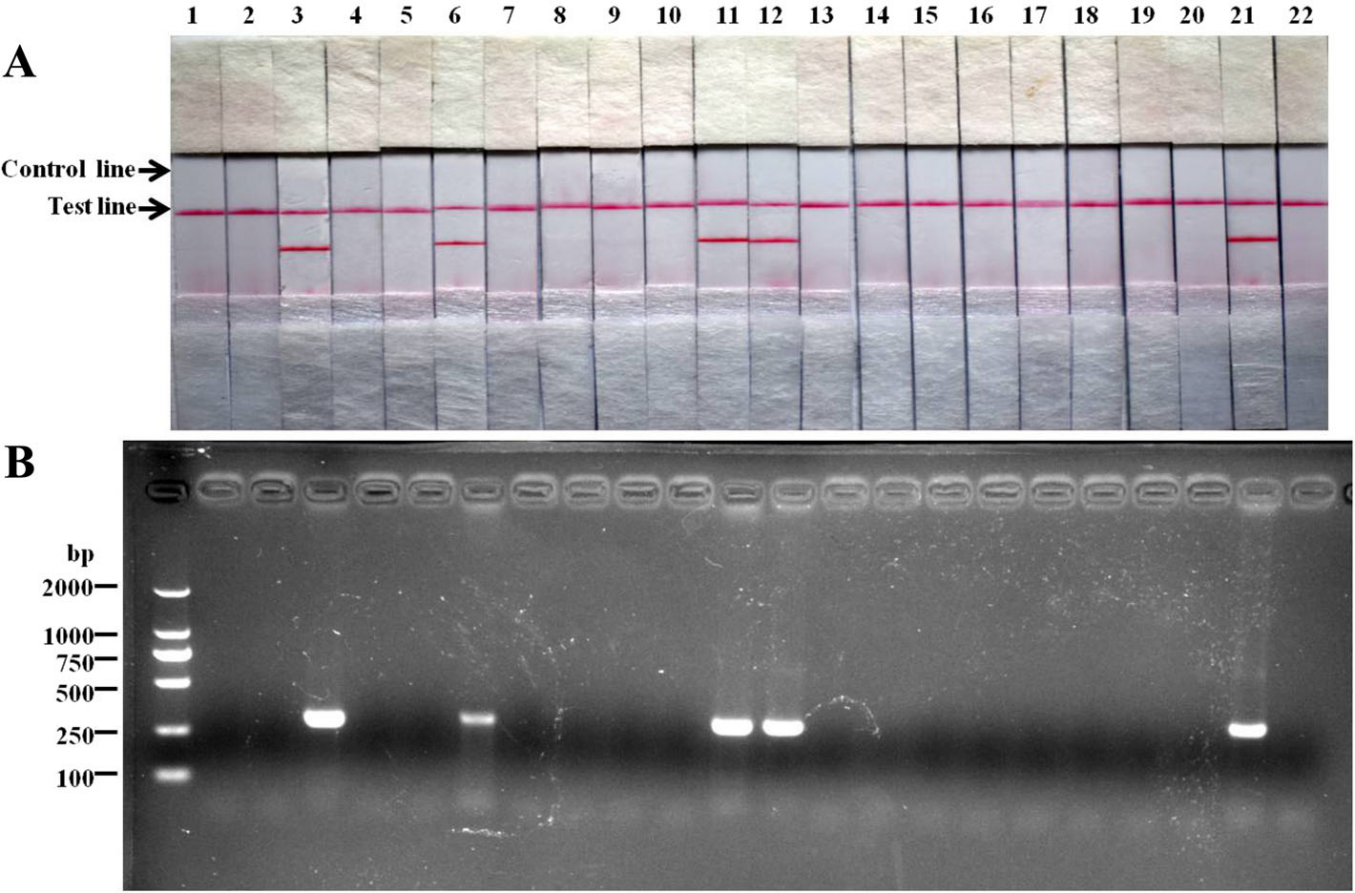
Representative detection of clinical fecal samples by RPA-LFD (**a**) and conventional PCR (**b**). Lane 1 to 20: clinical fecal samples; Lane 21: *L. intracellularis*-positive fecal sample; Lane 22: *L. intracellularis*-negative fecal sample

The clinical performance of the *L. intracellularis*-specific RPA-LFD was evaluated by using 150 clinical fecal samples from growing pigs suffering from diarrhea and poor performance. At the same time, the samples were tested by using conventional PCR. For each experiment, a *L. intracellularis* positive (fecal sample containing pure-cultured *L. intracellularis* from a commercial vaccine) and a negative control (fecal sample from *L. intracellularis*-negative healthy pigs detected by PCR) were included to ensure the test would not report a false positive. As shown in Fig. 4 and Table 2, the *L. intracellularis*-specific RPA-LFD could efficiently detect the *L. intracellularis* in clinical feces, and the detection results were in 100% coincidence with those of conventional PCR.

**Table 2:** Coincidence rate of RPA-LFD and conventional PCR

		PCR			CR
		Positive	Negative	Total	
RPA-LFD	Positive	90	0	90	
	Negative	0	60	60	100%
	Total	90	60	150	

*CR* Coincidence rate. CR = [(90 + 60) /150] × 100%

## Discussion

*L. intracellularis* is one of the most economically important pathogens in the swine production. It causes chronic diarrhea and poor performance in young growing pigs. Simple, rapid, sensitive and accurate methods suitable for field detection of *L. intracellularis* are crucial to efficiently control the infection and the spread of the *L. intracellularis* in time. Diagnosis of *L. intracellularis* is usually based on the demonstration of the microbe or microbial DNA in tissue specimens or fecal samples by PCR-based methods, or the detection of *L. intracellularis*-specific antibodies in sera by serological assays. However, the current diagnostic methods have several unneglectable limitations, such as antigen availability, the need of trained personnel to conduct the diagnostics, handling of the complex equipment, shipment and storage of samples to a distant laboratory. RPA is relatively new isothermal amplification method with the support of recombinase, a strand-displacement polymerase and a single-strand binding protein. The RPA does not need special instruments and can amplify target DNA to detectable level in less time and at lower temperature than those of other DNA amplification techniques. A lateral flow dipstick (LFD) is a visual testing tool that eliminates the need for trained personnel and expensive equipments. Therefore, the platform composed by RPA and LFD shows multiple advantages, such as convenient operation, rapid reaction, easy visualization of result and less equipments and skilled personnel requirement, which make it ideal for field diagnosis of *L. intracellularis* infection.

*L. intracellularis* is an obligate intracellular bacterium that is difficult to culture, and its detection and quantification pose a challenge for epidemiological and infection studies [16]. In this study, a rapid RPA-LFD assay was developed as an alternative diagnostic method of *L. intracellularis* for the first time. Two labelled primers targeting the dnaA gene of *L. intracellularis* were designed. The RPA reaction generated a dual-tagged DNA amplicon which could be visualized on the LFD with naked eyes by untrained personnel. This is especially important for areas where instruments and trained veterinary workers are deficient. Regarding the specificity of this RPA-LFD method, there was no cross-reaction with other usual porcine gastrointestinal bacteria and viruses. The sensitivity of the RPA-LFD was evaluated and compared with conventional PCR by using pure-cultured number-determined *L intracellularis* in this study. Since there is currently no gold standard method for the detection of *L. intracellularis*, and *L. intracellularis* cannot be cultured on artificial bacteriological media, it is hard to document accurately the specificity and sensitivity of the RPA test without samples confirmed by a gold standard test or bacteria isolation and identification. However the RPA-LFD could discriminate *L.intracellularis* from other common porcine gastrointestinal pathogens. Based on this result, the specificity of the RPA-LFD is 100%, even only one known *L.intracellularis* positive sample was included. Our results showed that the established RPA-LFD had a same detection threshold of 400 *L. intracellularis* per reaction for both pure-cultured *L. intracellularis* and feces sample spiked with pure-cultured *L. intracellularis*. Moreover, the RPA-LFD was found as sensitive as the conventional PCR. We believe that samples containing more than 400 *L. intracellularis* can be 100% detected by the RPA-LFD. To mimic the field detecting condition and to test whether fecal-PCR inhibitors were affecting RPA amplification and LFD detection, we also evaluated the sensitivity of the RPA-LFD by using DNA extracted from feces sample spiked with pure-cultured number-determined *L. intracellularis* as templates for RPA-LFD and conventional PCR. The detection limit of the RPA-LFD turned out to be 400 *L. intracellularis* per reaction in both cases. However, the specific bands amplified in conventional PCR from spiked feces samples were fainter than that of amplified from samples containing same number pure-cultured *L. intracellularis* (Fig. 3B). The above results suggest a decreased susceptivity to inhibition in RPA-FLD from feces in contrast to a greater susceptivity to inhibition in conventional PCR. Previous study has demonstrated that disease symptoms corresponded with higher fecal shedding of *L. intracellularis* detected by a SYBR green quantitative polymerase chain reaction (qPCR) assay after inoculating *L. intracellularis*-free pigs with 5 × 10^5^ bacteria per pig [20]. Thus, the sensitivity in term of number of *L. intracellularis* in feces sample presented in this study provided a means to monitor *L.intracellularis* shedding from naturally or experimentally infected pigs. Given the sensitivity of RPA-LFD was comparable to that of conventional PCR in detecting clinical feces samples, the RPA-LFD still had several advantages over the conventional PCR, such as more convenient operation, more rapid reaction, less equipment requirement and the results could be visualized by naked eyes.

The additional advantages of the *L. intracellularis* RPA-LFD included the elimination of the requirement of designing a special labelled probe, the shorter incubation time and lower single incubation temperature. Our study showed that the RPA-LFD assay could efficiently detect *L. intracellularis* from clinical fecal samples using two labelled primers rather than two primers and one probe in conventional RPA. The RPA-LFD assay could amplify target DNA at a relatively low, constant temperature from 25 to 42 °C, whereas the optimum reaction temperature was 30 to 42 °C. This means that a simple heating device, such as water bath or even body heat, can be used to achieve amplification and detection. The RPA-LFD assay could amplify the target DNA to detectable levels within 5 to 10 min, which was much shorter than those of conventional PCR-based methods. Currently, most of the reported RPA assays were developed by using commercial RPA kit from the TwistDx Company. The products of commercially available TwistAmp™ nfo kit can be detected by the LFD. However compatible probe is needed for the TwistAmp™ nfo kit. A TwistAmp™ LF probe consist of an oligonucleotide homologous to the target sequence which incorporates a 5’ -antigenic label, an internal abasic nucleotide analogue (a tetrahydrofuran residue or THF) which replaces a conventional base found in the target, and a polymerase extension blocking group (such as a phosphate, C3-spacer or a dideoxynucleotide) at the 3’ end, and the probe is typically 46–52 nucleotides long, at least 30 of which are placed 5’ to the THF site, and at least a further 15 are located 3’ to it [29]. Though the probe enhances the specificity of the RPA amplification, the probe is always hard to be found in a target gene and is expensive to be synthesized. Real-time loop-mediated isothermal amplification (RT-LAMP) has been developed for the detection of *L. intracellularis* [36]. However, the *L. intracellularis* RPA-LFD was faster than RT-LAMP (15 min in *L. intracellularis* RPA compared to 30–60 min in RT-LAMP). Moreover, RPA-LFD in this study only required a single pair of primers, while RT-LAMP needed at least 6 primers.

The diagnostic validation of the *L. intracellularis* RPA-LFD assay was evaluated with 150 clinical fecal samples and compared it with PCR. The reason we chose fecal samples is that *L. intracellularis* was always shed from feces of sick swine and the feces were easily acquired and non-invasive. As the sensitivity of RPA-LFD was comparable to that of conventional PCR in detecting clinical feces samples, the RPA-LFD results were in 100% coincidence with those of conventional PCR. Even though the conventional PCR is also suitable to detect *L. intracellularis*, the RPA-LFD still have multiple unique advantages as stated above.

In this study, the RPA templates could be prepared from clinical fecal samples with magnetic bead-based extraction methods. Compared with the traditional nucleic acid preparation methods, extraction using magnetic beads is easy and time saving, and does not require potentially dangerous procedures or specialized laboratory equipments [37]. The whole sample preparation and nucleic acid isolation process could be finished in 30 min. The combining the RPA-LFD with magnetic bead-based nucleic acid extraction techniques makes *L. intracellularis* on-site detecting possible.

## Conclusion

In summary, the established *L. intracellularis* RPA-LFD demonstrated multiple advantages, such as high sensitivity, good specificity, convenient operation, rapid reaction and less equipment requirement, which was suitable not only for pen side diagnosis but also for field screening and monitoring of the *L. intracellularis* infection. This technique can be used for epidemiological surveillance activities for *L. intracellularis*.

## Methods

### Primer design

Based on the published genome sequence of *L. intracellularis* PHE/MN1–00 strain (GenBank accession no. AM180252), a pair of specific primers targeting the dnaA (ATPase involved in DNA replication initiation) gene was designed for RPA: 5′- AAATCCAAAAGTCGAGTATCTAACTGCGG-3′; reverse primer: 5′- TAAAAACCCAGAGCAAAATCGTGATACCAGGCG-3′. The size of amplicon is 292 bp. In order to detect the RPA products by the LFD assay, the forward and reverse primers were labelled at the 5′-end with Fluorescein isothiocyanate (FITC) and Biotin, respectively.

### Nucleic acid extraction

Genomic DNA/RNA was extracted from *L. intracellularis* (a commercial live attenuated *L. intracellularis* vaccine, Enterisol®ileitis, Boehringer Ingelheim Vetmedica, Germany) and *Escherichia coli* (*E. coli*), *Salmonella* Cholerasuis (*S*. Cholerasuis), *Enterococcus faecalis* (*E. faecalis*), *Brachyspira hyodysenteriae* (*B*. *hyodysenteriae*) (ATCC-27164), porcine circovirus serotype 2 (PCV2), pseudorabies virus (PRV) Bartha-K61 strain, porcine rotavirus (PoRV), porcine epidemic diarrhea virus (PEDV) CV777 strain, transmissible gastroenteritis virus (TGEV) PUR46-MAD strain, classical swine fever virus (CSFV) vaccine C strain, samples containing the above pathogens except for *L. intracellularis* and samples containing all the above pathogens by using the genesig® DNA/RNA Extraction Kit (Primerdesign Ltd., United Kingdom) according to the manufacturer’s instructions. Briefly, 200 μl of sample were mixed well with 200 μl of lysis buffer and 20 μl of Proteinase K solution in a microcentrifuge tube. Then 500 μl magnetic beads/binding buffer were added to the lysed sample, mixed well and then separated by using a magnetic separator. The separated beads were washed by using Wash Buffer 1 and 2 and 80% ethanol, respectively. Add 50 μl Elution Buffer to the tube and resuspend the beads completely by repeated pipetting up and down. Then separate the magnetic beads from the sample and transfer the supernatant containing the purified DNA/RNA to a new tube and used for RPA. The protocol for extracting total DNA/RNA from clinical fecal samples is as follows: add 500 μl of Sample Prep Solution to approximately 10–20 mg of feces (a match head in size) or 200 μl if using liquid feces, and mix vigorously by shaking for 1 min. Allow the particles to settle down. Use up to 200 μl supernatant for the above extraction protocol.

### RPA basic reaction

RPA basic reaction was performed to test the usability of the primers with the TwistAmp Basic kit (TwistDx, UK) according to the manufacturer’s instruction. Briefly, the RPA reaction with a final reaction volume of 50 μl comprised of 29.5 μl rehydration buffer, 12 μl ddH_2_O, 2 μl of each primer (10 μM) and 2 μl template. To initiate the reaction 2.5 μl of magnesium acetate (280 mM) was added. Vortex and flip the reaction tube by hand or spin the reaction tube in a palm centrifuge. Reactions were performed at 37 °C in a water bath for 25 min. The amplification products were purified with QIAquick PCR Purification Kit (Qiagen, Germany) and analyzed on 2% agarose gel electrophoresis. For each experiment, a *L. intracellularis* negative (nucleic acid extracted from clinical feces which were negative for *L. intracellularis* determined by conventional PCR) and a blank control were included.

### RPA-LFD assay

For lateral flow dipstick detection of the RPA products, the forward and reverse primers for RPA were labelled at the 5′-end with FITC and Biotin, respectively. Different concentration of primers, reaction duration ranging from 5 min to 30 min, and reaction temperatures ranging from 37 °C to 42 °C were evaluated to optimize the condition for the RPA-LFD assay. After amplification, the RPA products were detected by lateral flow strip (Ustar Biotechnologies, Hangzhou, China) according to the instruction of manufacturer. The lateral flow strip contains streptavidin-coated colloidal gold on the sample pad, anti-FITC antibody on the Test line and biotin on the Control line. The result was considered valid if a pink line at the Control line was visible. The result was considered to be positive when the both Control line and pink Test line appeared.

### Evaluation of RPA-LFD

Based on the optimized conditions for RPA-LFD, the sensitivity of the RPA-LFD was evaluated and compared with that of the conventional PCR by using genomic DNA extracted from pure-cultured and number-determined (10^7^, 10^6^, 10^5^, 10^4^, and 10^3^ bacteria) *L. intracellularis* attenuated vaccine strain B3903 (a commercial live attenuated *L. intracellularis* vaccine, Enterisol®ileitis, Boehringer Ingelheim Vetmedica, Germany). To mimic the field detecting condition, the sensitivity of the RPA-LFD was also evaluated and compared with that of the conventional PCR by using genomic DNA extracted from feces sample spiked with pure-cultured and number-determined *L. intracellularis* (10^7^, 10^6^, 10^5^, 10^4^, and 10^3^ bacteria). The extracted DNA was dissolved in 50 μl Elution buffer, and 2 μl of extracted genomic DNA was used as templates for RPA-LFD and conventional PCR, respectively. Then the number of *L. intracellularis* per reaction was calculated as follow: bacterium number (10^7^–10^3^) / 50 μl × 2 μl, i.e. 4 × 10^5^–4 × 10^1^ bacteria per reaction. The PCR assay was performed in a 50 μl reaction containing 25 μl of 2 × Es T*aq* MasterMix (Beijing ComWin Biotech, Beijing, China), 2 μl of each primer (10 μM), 2 μl of template, 20 μl of ddH_2_O. The cycling parameters were 95 °C for 5 min, followed by 30 cycles of denaturation at 95 °C for 30s, annealing at 56 °C for 30s, extension at 72 °C for 20s, and a final extension of 72 °C for 10 min. The PCR was performed on a Bio-Rad T100 Thermal Cycler (Bio-Rad, USA). After amplification, the PCR products were analyzed using agarose gel electrophoresis; and the RPA products were detected by the LFD.

Since the most common porcine gastrointestinal pathogens including *E. coli*, *S*. Cholerasuis, *E. faecalis*, *B*. *hyodysenteriae*, PCV2, PRV,PoRV, PEDV, TGEV, and CSFV, are frequently detected in clinical feces from pigs with diarrhea in China, the specificity of the *L. intracellularis*-specific RPA-LFD was determined with DNA or RNA samples from these gastrointestinal pathogens. The 10 ng nucleic acids from each pathogen, samples containing the above pathogens except for *L. intracellularis* as well as a mixture of 10 ng nucleic acids from pure *L. intracellularis* and 10 ng nucleic acids from the above each gastrointestinal pathogens were respectively tested. The existence of each pathogen was demonstrated by conventional PCR using the primers in Table 1.

Clinical fecal samples from growing pigs suffering from diarrhea and poor performance were used to evaluate the diagnostic validity. For each experiment, a *L. intracellularis* positive (fecal sample containing pure-cultured *L. intracellularis* from a commercial vaccine) and a negative control (fecal sample from *L. intracellularis*-negative healthy pigs detected by PCR) were included to ensure the test would not report a false positive.

## Abbreviations

*B. hyodysenteriae*: *Brachyspira hydysenteriae*
CSFV: Classical swine fever virus
*E. faecalis*: *Enterococcus faecalis*
*E.coli*: *Escherichia coli*
ELISA: Enzyme-linked immunosorbent assay
IFA: Indirect immunofluorescence assay
IHC: Immunohistochemistry
IPMA: Immunoperoxidase monolayer assay
*L. intracellularis*: *Lawsonia intracellularis*
LFD: Lateral flow dipstick
PCR: Polymerase chain reaction
PCV2: Procine circovirus serorype2
PEDV: Porcine epidemic diarrhea virus
PoRV: Porcine rotavirus
PPE: Porcine proliferative enteropathy
PRV: Pseudorabies virus
qPCR: Quantitative polymerase chain reaction
RPA: Recombinase polymerase amplification
RT-LAMP: Real-time loop-mediated isothermal amplification
*S.*Cholerasuis: *Salmonella* Cholerasuis
TGEV: Transmissible gastroenteritis virus
